# Biomaterials and bioactive molecules to drive differentiation in striated muscle tissue engineering

**DOI:** 10.3389/fphys.2015.00052

**Published:** 2015-02-23

**Authors:** Valentina Di Felice, Giancarlo Forte, Dario Coletti

**Affiliations:** ^1^Department of Experimental Medicine and Clinical Neurosciences, University of PalermoPalermo, Italy; ^2^Dipartimento di Medicine e Terapie d'avanguardia, Strategie Biomolecolari e Neuroscienze, Istituto Euro-Mediterraneo di Scienza e TecnologiaPalermo, Italy; ^3^Integrated Center for Cell Therapy and Regenerative Medicine (ICCT), International Clinical Research Center, St. Anne's University HospitalBrno, Czech Republic; ^4^B2A Biological Adaptation and Ageing, Université Pierre-et-Marie-CurieParis, France; ^5^Department of Anatomical, Histological, Forensic Sciences and Hortopedics, Sapienza University of RomeRome, Italy; ^6^Interuniversity Institute of MyologyRome, Italy

**Keywords:** cardiac tissue engineering, regenerative medicine, scaffolds, vasculature niche, stem cell transplantation, skeletal muscle

The generation of engineered tissues and organs has entered into the clinical practice in response to the chronic lack of organ donors. In particular, for the skeletal and cardiac muscles the translational potential of tissue engineering approaches has clearly been shown, even though the construction of these tissues lags behind others given the hierarchical, highly organized architecture of striated muscles. Failure of the cardiac tissue leads to cardiovascular diseases, which are the leading cause of death in the developed world (Di Felice et al., 2014). On the other hand, there are many clinical cases where the loss of skeletal muscle due to a traumatic injury, an aggressive tumor, or prolonged denervation may be cured by the regeneration of the muscle tissue (Perniconi and Coletti, 2014).

In this volume, we have included articles from renowned researchers in the fields of skeletal and cardiac muscle engineering who have contributed with methods, original research, and review articles covering various aspects of native and synthetic biomaterials or three-dimensional (3D) structures able to induce stem cell differentiation and which may be used in pre-clinical and clinical studies.

Of the two bio-artificial systems described, one is a silicon chamber system for the generation of skeletal muscle constructs described by Snyman et al. (2013). This inexpensive and readily adaptable system (distance between pins, cell number, and matrix-cell volume can be readily changed) may be used with different hydrogels and applied to any existing culture chambers. The other is a PEG-fibrinogen (PF)-based hydrogel scaffold used to rejuvenate aged adult skeletal muscle-derived pericytes (MP) from pig skeletal muscle. In this 3D environment pericytes were able to recover their differentiation potential toward myogenic differentiation and vessel formation. Fuoco and colleagues demonstrated that the 3D PF environment was beneficial for swine-derived MP by mimicking the stiffness and mechanical properties of young muscle extracellular matrix (ECM), thus, “rejuvinating” aged MP may be an alternative to the use of myogenic progenitors; indeed, swine derived MP represent a valid alternative to build human size comparable artificial muscle units from the swine muscle (Fuoco et al., 2014).

The method of choice to seed stem cells in 3D cultures is important, in order to form a densely packaged proto-tissue, independently from the scaffold used. This step is fundamental for the cells of the proto-tissue to complete their maturation process and interconnect with the native tissue upon *in vivo* implantation. Pagliari et al. (2014a) showed a method for sequential cell seeding, where first gelatin scaffolds were colonized with human mesenchymal stem cells in a static condition to favor endothelialization; then the scaffolds were loaded with pre-committed cardiac progenitors and cultured in perfusion bioreactor in cardiogenic conditions. The authors obtained a well-packed cardiac proto-tissue, rich in vessels, but without functional contractile or vascular structures. They concluded that even the more complex 3D dynamic culture system needs to be improved with stretching or electric stimulation to obtain mature, functional cardiac tissue.

A valid alternative to biosynthetic scaffolds are de-cellularized organs, for example the muscle acellular scaffold (MAS) from skeletal muscle. Perniconi et al. (2014) demonstrated that MAS may be used to induce the differentiation of cultured myoblasts and this is an excellent support for 3D myogenic cultures also *in vivo* in orthotopical engraftments. MAS is histocompatible, porous, degradable and non-toxic. This structure is stable in anatomical sites other than the skeletal musculature, but does not provide enough signals to trigger myogenesis by the colonizing cells. The possibility to use MAS for the reconstruction of tissue different from the skeletal muscle should be investigated. For example, considering similarities between the skeletal and the cardiac tissue, MAS might be used as an autologous native scaffold for cardiac tissue transplantation.

The same authors also contributed a review article on the usefulness of the MAS as a scaffolding platform able to re-create the natural structure of the muscle. MAS is naturally embedded with active native molecules which remain active after the decellularization process and help the implanted proto-tissue to integrate in the host organ. Tissue-derived ECM helps in structuring niches rich in adhesive and signaling molecules supporting stem cells self-renewal and differentiation (Teodori et al., 2014).

When the muscle lesion is so extensive that the function of the muscle is impaired, the definition of volumetric muscle losses (VMLs) applies. In this case the muscle should be replaced by devices able to re-establish the function of the musculature and which preserve force transmission and continuity of the architecture within the host tissues. Hence, Cittadella Vigodarzere and Mantero (2014) described the architecture of the native skeletal tissue, identifying all the single elements which should be taken into consideration in a skeletal muscle tissue engineering construct. They focused the attention on the vasculature structures of constructs and, since decellularized ECM promotes vascularization of the implanted construct, they concluded that decellularized scaffolds are ready for a clinical application on human.

A clinical example of the latter is described in an interesting review discussing the importance of angiogenesis and the pro-angiogenic and pro-myogenic effects of the vascular endothelial growth factor (VEGF)/VEGF receptor pathway as a therapeutic strategy to cure muscle weakness and cardiomyopathy in Duchenne muscular dystrophy (DMD) patients (Shimizu-Motohashi and Asakura, 2014).

Apart from the structures of ECM and vasculature, also growth factors, cytokines, pleiotropic signaling pathways, and cell-specific regulators play an important role in the differentiation and self-renewal of cardiac as well as skeletal muscle stem cells. Pagliari et al. (2014b) described the most important signaling pathways which influence the *in vivo* differentiation of cardiac progenitor cells. Many of these pathways and factors have different and distant effects on the differentiation process (JAK/STAT, Hippo pathway, Wnt and Notch signaling, etc.).

This book provides a comprehensive up-to-date review on cardiac and skeletal muscle tissue engineering and highlights the several elements which have to be taken into consideration when engineering a functional proto-tissue.

## Conflict of interest statement

The authors declare that the research was conducted in the absence of any commercial or financial relationships that could be construed as a potential conflict of interest.
